# Novel *KCNQ4* variants in different functional domains confer genotype- and mechanism-based therapeutics in patients with nonsyndromic hearing loss

**DOI:** 10.1038/s12276-021-00653-4

**Published:** 2021-07-28

**Authors:** Sang-Yeon Lee, Hyun Been Choi, Mina Park, Il Soon Choi, Jieun An, Ami Kim, Eunku Kim, Nahyun Kim, Jin Hee Han, Min young Kim, Seung min Lee, Doo-Yi Oh, Bong Jik Kim, Nayoung Yi, Nayoung, K. D. Kim, Chung Lee, Woong-Yang Park, Young Ik Koh, Heon Yung Gee, Hyun Sung Cho, Tong Mook Kang, Byung Yoon Choi

**Affiliations:** 1 Department of Otorhinolaryngology-Head and Neck Surgery, Seoul National University Hospital Seoul National University College of Medicine, Seoul, Korea; 2 Department of Otorhinolaryngology-Head and Neck Surgery, Seoul National University Bundang Hospital Seoul National University College of Medicine, Seongnam, Korea; 3 Department of Physiology, Sungkyunkwan University School of Medicine, Samsung Medical Center, Suwon, Korea; 4 Department of Otorhinolaryngology-Head and Neck Surgery, Seoul Medical Center, Seoul, Korea; 5 Stem Cell & Regenerative Medicine Institute, Samsung Medical Center, Seoul, Korea; 6 Department of Otolaryngology and Head & Neck Surgery, Chungnam National University College of Medicine, Daejeon, Korea; 7 Samsung Genome Institute, Samsung Medical Center, Seoul, Korea; 8 Department of Pharmacology, Brain Korea 21 PLUS Project for Medical Sciences, Yonsei University College of Medicine, Seoul, Korea; 9 Department of Anesthesiology and Pain Medicine, Samsung Medical Center, Sungkyunkwan University School of Medicine, Seoul, Korea

**Keywords:** Genetics research, Translational research

## Abstract

Loss-of-function variant in the gene encoding the KCNQ4 potassium channel causes autosomal dominant nonsyndromic hearing loss (DFNA2), and no effective pharmacotherapeutics have been developed to reverse channel activity impairment. Phosphatidylinositol 4,5-bisphosphate (PIP_2_), an obligatory phospholipid for maintaining KCNQ channel activity, confers differential pharmacological sensitivity of channels to KCNQ openers. Through whole-exome sequencing of DFNA2 families, we identified three novel *KCNQ4* variants related to diverse auditory phenotypes in the proximal C-terminus (p.Arg331Gln), the C-terminus of the S6 segment (p.Gly319Asp), and the pore region (p.Ala271_Asp272del). Potassium currents in HEK293T cells expressing each KCNQ4 variant were recorded by patch-clamp, and functional recovery by PIP_2_ expression or KCNQ openers was examined. In the homomeric expression setting, the three novel KCNQ4 mutant proteins lost conductance and were unresponsive to KCNQ openers or PIP_2_ expression. Loss of p.Arg331Gln conductance was slightly restored by a tandem concatemer channel (WT-p.R331Q), and increased PIP_2_ expression further increased the concatemer current to the level of the WT channel. Strikingly, an impaired homomeric p.Gly319Asp channel exhibited hyperactivity when a concatemer (WT-p.G319D), with a negative shift in the voltage dependence of activation. Correspondingly, a KCNQ inhibitor and chelation of PIP_2_ effectively downregulated the hyperactive WT-p.G319D concatemer channel. Conversely, the pore-region variant (p.Ala271_Asp272del) was nonrescuable under any condition. Collectively, these novel KCNQ4 variants may constitute therapeutic targets that can be manipulated by the PIP_2_ level and KCNQ-regulating drugs under the physiological context of heterozygous expression. Our research contributes to the establishment of a genotype/mechanism-based therapeutic portfolio for DFNA2.

## Hearing loss: genetically tailored treatment strategy for congenital deafness

People with a certain type of inherited hearing loss may stand to benefit from a personalized, genetically tailored treatment strategy. A research team from South Korea led by Byung Yoon Choi from Seoul National University College of Medicine identified three new mutations in *KCNQ4*, a gene that encodes a potassium channel protein found in the inner ear, that can cause congenital deafness. Strikingly, the authors identified the first hyperactive *KCNQ4* variant. They inserted these gene variants into human cells in culture, and found that drugs known to affect the activity of this channel protein had different effects on different mutations. Impairments caused by two of the variants were prevented by drug treatment; the third variant proved resistant to the same therapeutic approach. The authors propose further validating the therapeutic responsiveness of different *KCNQ4* variants in genetically engineered mice.

## Introduction

The *KCNQ4* (Kv7.4) gene, which encodes a voltage-gated potassium channel protein, can cause autosomal dominant nonsyndromic hearing loss (DFNA2) when mutated^1–4^, accounting for approximately 9% of all ADNSHL cases^5^. To date, over 30 variants have been shown to cause nonsyndromic sensorineural hearing loss (NSHL)^5–8^. KCNQ4 is highly expressed in the basolateral membrane of outer hair cells (OHCs) in the cochlea and is involved in the formation of M-type potassium currents that repolarize the cells, reduce cell excitability, and regulate many physiological responses^1,9^. KCNQ4 plays an essential role in the recycling of potassium ions (K^+^), allowing for sound transduction and maintenance of the resting membrane potential and osmotic equilibrium^10^. Correspondingly, *kcnq4*^*−/−*^ mice primarily show degeneration of OHCs and progressive hearing loss, mirroring DFNA2 in humans^11^.

The KCNQ4 protein comprises six transmembrane domains (S1–S6), namely, four voltage-sensor domains (S1−S4) and a pore region (S5, pore loop, and S6); the N- and C-termini are intracellular^6^. As KCNQ4 channels form homo- and heteromeric assemblies of the four pore-forming subunits, variants in a single subunit may disrupt the channel function of the tetramer *via* dominant-negative inhibition^12^. The majority of *KCNQ4* variants responsible for DFNA2 are clustered in the S5−S6 region, surrounding the ion permeating pore region (amino acids 271−292). Pore variants, particularly in the potassium ion selectivity filter with a signature GYG motif or core pore domain are closely associated with loss-of-function; disruption of ion permeation, independent of channel gating, and dominant-negative inhibitory effects have been suggested as the pathogenesis underlying DFNA2^1^.

Voltage-gated ion channels are promising drug targets. The most popular strategies for modulating voltage-gated ion channel function with small molecules have been targeted at voltage-sensor domain (VSD) activation, pore domain (PD) opening, or permeation^13^. To the best of our knowledge, only channel openers have been reported as potent molecules for restoring impaired KCNQ4 channel activity. However, channel openers primarily modify gating kinetics rather than ion conductance^12^. Thus, KCNQ4 mutant channels containing pore variants tend to be less amenable to the pharmacological application of channel openers.

Recently, VSD-PD coupling has emerged as a potential pharmacological target, regulating a current without significantly changing the critical properties of voltage dependence and time dependence^14^. In addition, the phospholipid phosphatidylinositol 4,5-bisphosphate (PIP_2_) has been reported to act as an obligate ligand that activates KCNQ channels by regulating the VSD-PD coupling state^15^. More specifically, defective residues in potential PIP_2_-binding sites in KCNQ channels have been reported to affect gating kinetics and regulate the specificity of tetramer assembly^13,16,17^, leading to various human phenotypes, such as long QT syndrome and epilepsy. Moreover, a structural target for PIP_2_ binding related to VSD-PD coupling has been revealed, as evidenced by cryo-EM-based KCNQ structures, including KCNQ4^18^; thus, PIP_2_ regulation may contribute to the development of DFNA2 therapeutics.

Here, we introduce two novel DFNA2-causing missense *KCNQ4* variants located far distal to the essential pore-loop region, where most loss-of-function variants occur. The p.Gly319Asp (p.G319D) variant involves the C-terminal part of the transmembrane S6 segment; p.Arg331Gln (p.R331Q) is in the proximal part of the cytoplasmic C-terminus, which has been considered a potential PIP_2_-binding domain. Considering that PIP_2_ binding guarantees KCNQ4 channel activity and regulates pharmacological sensitivity to KCNQ channel openers, these variants are potential targets for pharmacological therapy to restore lost function.

## Materials and Methods

### Subjects

All procedures in this study were approved by the Institutional Review Boards of Seoul National University Hospital (IRB-H-0905-041-281) and Seoul National University Bundang Hospital (IRB-B-1007-105-402). Four families (SB228, SB155, SB356, and SB62) were enrolled. The affected individuals underwent comprehensive phenotypic evaluations, including medical history interviews, physical examinations, imaging, and audiological assessments.

### Molecular genetic testing and diagnosis

As previously described^19,20^, exome sequencing was performed followed by bioinformatics analyses. Rare single-nucleotide variations, indels, or splice-site variants were chosen using a comprehensive filtering process. The pathogenic potential of novel variants was evaluated according to the American College of Medical Genetics and Genomics/Association for Molecular Pathology (ACMG/AMP) guidelines^21,22^.

### Plasmid construction, cell culture, and transfection

The WT *KCNQ4* cDNA was cloned into the SgfI and KgockI sites of pEGFP (enhanced green fluorescent protein)-N1. The monkey embryonic kidney cell line COS-7 (Korea Culture Line Bank, Korea) was maintained in Dulbecco′s modified Eagle′s medium (DMEM) with 10% fetal bovine serum. Before transient transfection, the cells were plated at a density of 70–80% confluency in a lab Tek II chamber. The cells were transfected with a plasmid expressing wild-type or mutant *KCNQ4* using Lipofectamine 3000 (Invitrogen, Seoul, Korea) and incubated at 37 °C for 24 hours. The cells were then stained with concanavalin A^23^.

### Cell culture and transfection of KCNQ4 plasmids for electrophysiology

HEK293T cells were maintained in DMEM (Thermo Fisher) supplemented with 10% fetal bovine serum (Thermo Fisher) and 1% penicillin/streptomycin (Thermo Fisher) at 37 °C in 5% CO_2_. The cells were subcultured to 70–90% confluence and plated for transfection. A day before transfection, the cells were plated to 70–90% confluence in a 6-well culture dish. To record whole-cell ion currents of homotetrameric KCNQ4 channels, *KCNQ4* wild-type (WT), p.Ser269del (p.S269del), p.Ala271_Asp272del (p.A271_D272del), p.G319D, or p.R331Q was cloned into the pRK5 vector, and each plasmid (4.0 μg) was transiently expressed with pEGFPN-1 (0.4 μg, BD Biosciences) in HEK293T cells using Lipofectamine 2000 (Invitrogen). Tandem concatemers of KCNQ4 WT subunits or of one WT and one mutant subunit were generated by fusing the subunit C-terminus to the N-terminus. Five tandem concatemers fused with *KCNQ4* WT (WT-WT, WT-p.S269del, WT-p.A271_D272del, WT-p.G319D, and WT-p.R331Q) were cloned in the pRK5 vector, and each (8.0 μg) was transiently expressed in HEK293T cells with pEGFPN-1 (0.4 μg). HEK293T cells transfected with the empty pRK5 and GFP vectors were used as a nontransfected control group (GFP). The cDNA of phosphatidylinositol 4-phosphate 5-kinase (PIP5K) was kindly gifted by Dr. Insuk So (Seoul National University, Korea) and cloned into the pEGFP vector, which was transiently expressed in HEK293T cells with *KCNQ4* plasmid. One day after transfection, cells were detached by 0.05% trypsin/EDTA (Thermo Fisher), and whole-cell patch-clamp analysis was performed.

### Whole-cell patch-clamp

KCNQ4 channel currents were recorded with a conventional whole-cell patch-clamp. Patch pipettes were pulled from borosilicate glass tubing (WPI, Sarasota, FL, USA), and the pipette tip was fire polished with a microforge (MF-83; Narishige, Japan). The final pipette tip resistance, when filled with pipette solution, was 1.5–3 MΩ. Whole-cell K^+^ currents were recorded with an Axopatch-1D amplifier (Axon Instrument, USA). The currents were filtered at 5 kHz and acquired at a sampling rate of 10 kHz. Before acquiring ionic currents, the series resistance was compensated, and the cell membrane capacitance (C_m_) was measured and canceled using a circuit of the patch-clamp amplifier. The K^+^ currents were generated with 2-s depolarizing voltage steps, ranging from −70 to +40 mV in 10-mV increments, followed by a 1-s hyperpolarizing voltage step to −50 mV. Steady-state activation curves were constructed to analyze the voltage-dependent gating of KCNQ4 channels. To construct steady-state activation curves, KCNQ4 channels were activated by 2-s depolarizing prepulse voltage steps (from −90 to +40 mV in 10-mV increments), and then tail currents were generated by a 0.5-s repolarizing voltage step to −40 mV. The measured peak tail current amplitudes were plotted against activating prepulse voltages, and they were fitted by the Boltzmann function to calculate half-activation voltages (V_0.5_). If necessary, V_0.5_ values were obtained from I-V curves by calculating the normalized conductance (G/G_max_). The KCNQ4-mediated K^+^ current from the total whole-cell current was pharmacologically isolated by treating cells with a KCNQ inhibitor (linopirdine, 30 μM), and the linopirdine-sensitive component was obtained by digital subtraction. For neutralization of the negative charges of membrane PIP_2_, poly-L-lysine (PLL, 10 or 30 μg/ml)—a polycationic agent—was included in the whole-cell pipette solution before achieving the conventional whole-cell configuration. For comparison, KCNQ4 current amplitudes recorded at +40 mV were divided by the measured C_m_, and the results are expressed as current densities (pA/pF). All patch-clamp recordings were performed at room temperature (~23 °C). The recorded currents were analyzed using Clampex software (pCLAMP 7.0; Axon Instrument). Whole-cell currents for HEK293T cells transfected with the empty pRK5 and GFP vectors were used as the negative control.

### Chemicals and solutions for electrophysiology

The external bath solution for whole-cell voltage-clamp recording consisted of 147 mM NaCl, 5 mM KCl, 1.5 mM CaCl_2_, 1 mM MgCl_2_, 10 mM HEPES, and 10 mM D-glucose, adjusted to pH 7.4 with N-methyl-D-glucamine (NMDG). The internal patch pipette solution contained 130 mM KCl, 10 mM NaCl, 10 mM EGTA, 10 mM HEPES, 3 mM Mg-ATP, and 0.5 mM CaCl_2_, adjusted to pH 7.2 with KOH. The calculated free Ca^2+^ concentration was ~10 nM. Linopirdine dihydrochloride (Tocris Bioscience, Bristol, UK), retigabine (Glentham Life Science, Corsham, UK), ML213 (Tocris Bioscience, Bristol, UK), and zinc pyrithione (Sigma-Aldrich, St. Louis, MO, USA) were dissolved in DMSO and prepared as 1000x stock (5-30 mM) solutions. Each stock solution was diluted into the external bath solution before use.

### Evaluation of cellular trafficking of wild-type and mutant KCNQ4

After incubation, transfected cells were fixed in 4% paraformaldehyde for 15 min, followed by washing with PBS, which was repeated three times. The cells were incubated with primary antibodies [ANTI-FLAG (Sigma-Aldrich Corp., St. Louis, MO, USA)] at 24 °C for 160 min, washed three times with chilled (4 °C) PBS, and incubated with secondary antibodies [F(ab’)2-goat antimouse IgG (H + L), Invitrogen, Seoul, Korea] at room temperature for 90 min. The samples were mounted with VECTASHIELD mounting medium (Vector Laboratories, CA, USA), and images were taken using a confocal microscope (Carl Zeiss, LSM710).

### Cell surface biotinylation assay

A total of 5.0 × 10^6^ COS-7 cells (in a T75 flask) were transfected with KCNQ4 WT and mutant plasmids (WT, p.R331Q and p.A271_D272del) using Lipofectamine Plus reagent (Life Technologies, Inc.) and incubated at 5% CO_2_ and 37 °C for 24 h. Cell surface proteins (expressed WT or mutant KCNQ4 proteins on the cell surface) were isolated using a PIERCETM cell surface protein isolation kit (Thermo Scientific) and analyzed with a Myc-tag (Abcam)-based ELISA kit (Thermo Scientific).

### Statistical analysis

Data were plotted with Origin software (version 6.1, OriginLab), and the results are shown as the mean ± standard error of the mean (SEM), with *n* denoting the sample number. Statistical significance was determined by paired or unpaired Student’s *t*-test or ANOVA, and differences were considered significant at *P* < 0.05.

## Results

### Clinical phenotypes of four KCNQ4 variants

**Fig. 1:**
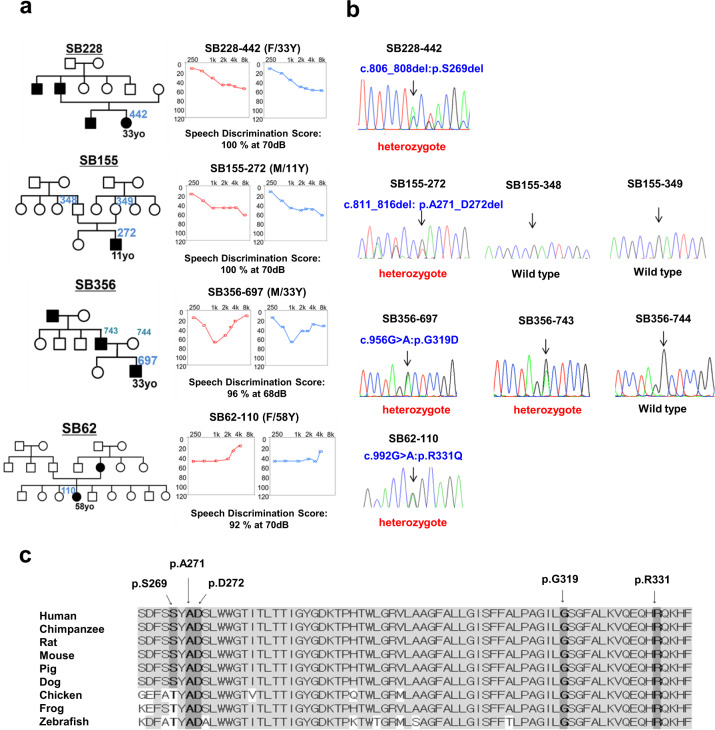
Pedigree and audiological phenotypes and Sanger sequencing traces of novel KCNQ4 variants and the diverse topology of all reported KCNQ4 variants segregating with DFNA2. **a** Pure-tone audiometry of the four probands with different audiological phenotypes: high-frequency (Families SB228 and SB155), mid-frequency (SB356), and low-frequency (SB62) sensorineural hearing loss. The upper horizontal axis shows tone frequency (Hz); the vertical axis indicates the hearing level (dB). We denote bone and air conduction pure-tone thresholds at different frequencies in the right (red color) and left (blue color) ears. **b** Pedigrees and Sanger sequence chromatograms. Three variants (p.A271_D272del (SB155), p.G319D (SB356), and p.R331Q of KCNQ4 (SB62)) are novel: a *de novo*, single heterozygous, in-frame deletion (SB155) and autosomal dominant, single heterozygous, missense variants of KCNQ4 (SB356 and SB62). **c** Well-conserved residues of three novel variants among various KCNQ4 orthologs.

Notably, diverse audiological configurations were observed in four DFNA2 families with segregating *KCNQ4* variants. Audiological configurations were classified into three types: high-frequency (Families SB228 and SB155), mid-frequency (SB356), and low-frequency (SB62) hearing loss (Fig. 1a). SNHL in families SB228, SB356, and SB62 was autosomal dominant; that in SB155 was sporadic (Fig. 1a). The age of onset for SNHL and the age of ascertainment for proband SB228-442 were in the early 20 s and at 33 years, respectively. The affected individuals in the SB228 family, including the proband’s sibling and father, developed high-frequency hearing loss in their late 20 s and at the time of the study were using hearing aids due to progressive hearing deterioration. In the SB155 family, proband SB155-272 initially exhibited minimally progressive symmetrical high-frequency hearing loss with a downsloping configuration at the age of 11 years (Fig. 1a). Contrary to family SB228, both parents in SB155 had normal hearing thresholds across all frequencies. In the SB356 family, proband SB356-697 (33 years of age at ascertainment) manifested moderate-to-severe hearing loss, displaying predominantly mid-frequency hearing loss (symmetric notch at 1 kHz up to 60 dB HL in both ears), namely, cookie-bite configuration (Fig. 1a). However, there was no change in hearing thresholds over a 1-year follow-up period in the affected individual. The hearing status of the proband’s father was allegedly poor but had not been documented. In the SB62 family, proband SB62-110 (58 years of age at ascertainment) manifested symmetrical low- and mid-frequency hearing loss of approximately 40 dB HL (Fig. 1a). The pedigree indicated an autosomal dominant inheritance of hearing loss. During the 10-year follow-up period, SB62-110 experienced a definite progressive deterioration of bilateral hearing across all frequencies, as well as decreased speech discrimination scores. The proband’s audiogram eventually showed hearing thresholds at all frequencies of 65 dB HL in both ears. The proband’s mother had also experienced hearing loss in both ears at the age of 60 years; however, a corresponding hearing test had not been performed. No cochleovestibular malformations were noted by radiological evaluation in the affected individuals.

### Identification of novel *KCNQ4* variants

We identified four potential causative variants of SNHL: three are novel (c.811_816del:p.A271_D272del (SB155), c.G956 > A:p.G319D (SB356), and c.G992 > A:p.R331Q of *KCNQ4* (SB62)) (Fig. 1b). The other is a previously reported in-frame deletion (c.806_808del:p.S269del) found in SB228^24,25^. Specifically, none of the biological parents in SB155 were phenotypically affected, nor did they carry p.A271_D272del, as evidenced by haplotype phasing from trio ES data^26,27^, thus indicating that the p.A271_D272del arose *de novo* as a causative variant in the SB155 family (Fig. 1b). As confirmed by haplotype phasing from trio ES data^26,27^, the biological parents in SB155 were not phenotypically affected or carriers of the variant. The novel in-frame deletion variant, p.A271_D272del is located in the pore-loop domain of KCNQ4 (Fig. S1), where the majority of known KCNQ4 variants are linked to the hearing loss cluster. This deletion of six bases (GCCGAC) results in the loss of alanine and aspartic acid. This deletion is not listed in Korean Reference Genome Database (KRGDB, 1722 individuals); global minor allele frequency, including in Exome Aggregation Consortium (ExAC) and Genome Aggregation Database (gnomAD) (http://gnomad.broadinstitute.org/), was not found. These residues are highly conserved among *KCNQ4* orthologs (http://genome.ucsc.edu/) (Fig. 1c), coupled with a high Genomic Evolutionary Rate Profiling (GERP ++) score of 5.08. This variant is consistently predicted as “disease-causing” through *in silico* analyses, including combined annotation-dependent depletion (CADD) (https://cadd.gs.washington.edu/). Confirmation of *de novo* occurrence of p.A271_D272del indicates “moderate evidence” for PS2 in relation to hearing loss. Moreover, PM1 can be applied because the variant p.A271_D272del is located in the KCNQ4 pore-forming region, as evidenced by a well-studied functional domain without a benign variant. Therefore, the variant p.A271_D272del may be classified as “likely pathogenic” based on the ACMG/AMP guidelines used to classify variants, including PS2_moderate, PM1, PM2, and PS3 supporting (Table 1).

**Table 1:** KCNQ4 variants in the current study and pathogenicity prediction analysis.

Family	Genomic position (GRCh37/hg19)	HGVS		Zygosity	Inheritance	*In silico* Prediction		Ethnicity MAF		Global MAF		ACMG/AMP 2018 guidelines	
		Nucleotide change	Amino Acid change			CADD	REVEL	GERP	KRGDB (1722 individuals)	ExAC	gnomAD	Criteria	Classification
SB228-442	Chr1:41285116-41285118	c.806_808del	p.Ser269del (p.S269del)	Het	Dominant	19.1	NA	5.08	Absent	Absent	Absent	PM1, PM2, PM4 PS3_supporting	Likely pathogenic
SB155-271	Chr1:41285121-41285126	c.811_816del	p.Ala271_ Asp272del (p.A271_ D272del)	Het	De novo*	22.7	NA	5.08	Absent	Absent	Absent	PS2_moderate, PM1, PM2, PM4, PS3_supporting	Likely pathogenic
SB356-697	Chr1:41285847	c.956 G > A	p.Gly319Asp (p.G319D)	Het	Dominant	29.7	0.938	5.27	Absent	Absent	Absent	PM2, PP3, PS3_supporting	VUS
SB62-110	Chr1:41285883	c.992 G > A	p.Arg331Gln (p.R331Q)	Het	Dominant	33	0.919	5.44	Absent	Absent	0.000004102/1	PM2 PP3, PS3_supporting	VUS

*MAF* minor allele frequency, *Het* heterozygote, *VUS* variant uncertain significance, *NA* not available.

Refseq transcript accession number NM_001174069.1; Refseq protein accession number NP_001167540.

HGVS: Human Genome Variation Society (https://www.hgvs.org/).

Sequence Variant Nomenclature (http://varnomen.hgvs.org/).

CADD: Combined Annotation Dependent Depletion (https://cadd.gs.washington.edu/).

REVEL: Rare Exome Variant Ensemble Learner (https://sites.google.com/site/revelgenomics/).

KRGDB: Korean Reference Genome Database (http://coda.nih.go.kr/coda/KRGDB/index.jsp).

ExAC: Exome Aggregation Consortium databases.

gnomAD: The Genome Aggregation Database (https://gnomad.broadinstitute.org/).

ACMG/AMP 2018 guideline (http://wintervar.wglab.org/).

*Note that the biological parents of SB155 were not phenotypically affected or carriers of the variant, as confirmed by haplotype phasing from trio exome sequencing data.

Another novel missense variant, c.G956 > A:p.G319D (SB356 family) in the distal end of the transmembrane S6 segment (Fig. S1) is highly conserved among *KCNQ4* orthologs (Fig. 1c), as supported by a high GERP ++ score of 5.27. The variant was not found in public databases. This variant is consistently predicted as “disease-causing” by *in silico* analyses, including CADD and rare exome variant ensemble learner (REVEL) (https://sites.google.com/site/revelgenomics/). Accordingly, p.G319D can be classified as a “variant of uncertain significance (VUS)” based on ACMG/AMP guidelines (Table 1).

**Fig. 2:**
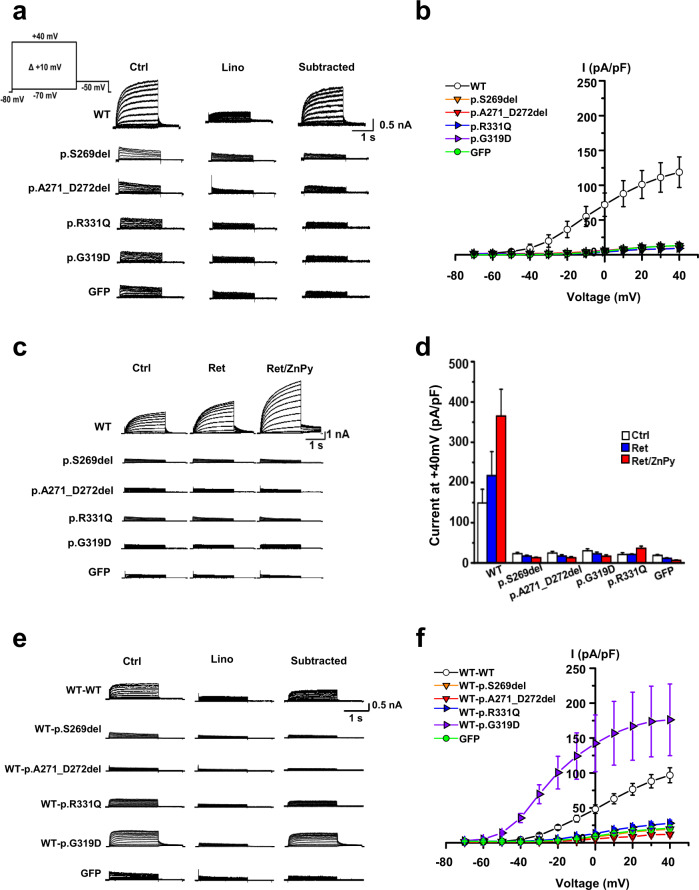
Impaired channel conductance and dominant-negative effects of KCNQ4 mutant channels. **a** Whole-cell K^+^ currents recorded from HEK293T cells transiently expressing KCNQ4 WT, p.S269del, p.A271_D272del, p.G319D, p.R331Q, or GFP. Linopirdine (30 μM)-sensitive K^+^ currents (‘subtracted’) from homomeric KCNQ4 mutant channels were barely detectable. **b** Current-voltage (I-V) relationships of linopirdine-sensitive K^+^ currents. **c** KCNQ openers did not activate homomeric KCNQ4 mutant channels. Retigabine (Ret, 10 μM) or a combination of Ret and zinc pyrithione (Ret/ZnPy 10 μM) did not activate mutant channels. **d** After treatment of KCNQ openers, the current densities of mutant channels were not significantly different from that of GFP (n = 7–9). **e** Representative K^+^ current traces recorded from KCNQ4 channels assembled from WT-WT, WT-p.S269del, WT-p.A271_D272del, WT-p.G319D, and WT-p.R331Q tandem concatemers. GFP-transfected cells were used as negative controls, and linopirdine-sensitive currents were subtracted for comparison. **f** I-V relationships of linopirdine-sensitive K^+^ currents of the concatemer channels (*n* = 4–21). Mean ± SEM.

The last novel missense variant, p.R331Q (SB62 family), is the first reported variant involving the proximal cytoplasmic C-terminus (Fig. S1). This residue is highly conserved among *KCNQ4* orthologs (Fig. 1c), as reflected by a high GERP++ score of 5.44. This variant has an extremely low MAF, satisfying PM2 criteria. CADD and REVEL show higher scores of 33 and 0.768, respectively, indicating “disease-causing” pathogenicity. Additionally, the mutant protein exhibited almost completely abrogated voltage-activated potassium currents compared with the WT protein (Fig. 2), supporting PS3. PP3 can also be applied due to a higher REVEL score of 0.8. Collectively, the p.R331Q variant was classified as “VUS” (Table 1).

### Effects of novel variants on KCNQ4 channel function

To test the effects of KCNQ4 variants on voltage-gated channel activity, we recorded whole-cell currents in HEK293T cells transiently transfected with plasmids expressing the WT and mutant proteins *via* patch-clamp recording. Whole-cell currents for HEK293T cells transfected with the empty pRK5 and GFP vectors were used as negative control; cells transfected with p.S269del KCNQ4, which is known to cause DFNA2, were used as a null-function control.

The linopirdine-sensitive potassium currents of the WT protein exhibited a typical KCNQ4 channel current, with voltage-dependent slow activation and outward rectification (Fig. 2a, b). Cells expressing the WT protein exhibited voltage-dependent potassium currents with a peak current density of 121.8 ± 25.4 pA/pF at +40 mV (*n* = 12). In contrast, the outward potassium currents generated by mutant channels (p.A271_D272del, p.R331Q, and p.G319D) were barely detectable, similar to the null-function and negative controls (Fig. 2b). Thus, our newly identified *KCNQ4* variants all showed complete loss of function in a homologous expression setting.

We further investigated whether the lost channel activity of these mutant proteins, especially in a homologous setting was restored by known KCNQ channel openers, such as retigabine (Ret, 10 μM), zinc pyrithione (ZnPy, 10 μM), or a combination of them (Ret/ZnPy). The combined application of channel openers (Ret/ZnPy) potentiated the KCNQ4-mediated potassium currents of the WT protein by more than twofold (Fig. 2c, d). However, homotetrameric channels containing the novel KCNQ4 variants (p.A271_D272del, p.R331Q, and p.G319D) displayed almost no potassium current, even with channel openers, and the currents were comparable to those in the null-function and negative controls.

### Dominant-negative effects of novel KCNQ4 variants

To better understand the pathogenesis of our novel KCNQ4 variants in a heteromeric system, we measured potassium currents in HEK293T cells coexpressing WT and each variant at various molar ratios (WT/variant ratios ranging from 4:0 to 0:4). The resulting KCNQ4-mediated potassium currents appeared to inversely correlate with the concentration of mutant cDNA (Fig. S2), without definite dominant-negative effects of the mutant proteins on the WT protein (peak current density of KCNQ4WT:vector (2:2): 131.7 ± 21.1 pA/pF at +40 mV, *n* = 11; peak current density of KCNQ4WT:KCNQ4p.A271_D272del (2:2): 69.8 ± 19.9 pA/pF at +40 mV, *n* = 7; peak current density of KCNQ4WT:KCNQ4p.G319D (2:2): 152.7 ± 45.7 pA/pF at +40 mV, *n* = 11; peak current density of KCNQ4WT:KCNQ4p.R331Q (2:2): 55.1 ± 17.8 pA/pF at +40 mV, *n* = 6). Except for the null-function control (p.S269del pore variant), the potassium currents generated by our novel mutant channels deviated from the predicted suppression ratios for tetrameric coassembly of WT with dominant-negative subunits. Interestingly, the KCNQ4WT:KCNQ4p.G319D-mediated current deviated significantly from the prediction and showed a larger current than the WT channel at 3:1 or 2:2 cDNA ratios. Nevertheless, we were unable to draw a definitive conclusion due to variance and outliers in current amplitudes within the same group (Fig. S2). Taken together, the results did not necessarily show that the novel KCNQ4 variants exerted a classical dominant-negative effect on functional WT channels, indicating the need for future studies using tandem concatemer channels.

Hence, we developed tandem concatemers by fusing each mutant protein with the functional WT protein under the same translational frame whereby translation of WT KCNQ4 precedes the KCNQ4 variant to reveal any dominant-negative inhibition under forced 2:2 assembly (i.e., homogenous heterotetramerization) (Fig. 2e, f). No defects in the synthesis of these concatemers were observed (data not shown). Potassium currents were barely detectable for the WT-p.A271_D272del concatemer channel, similar to those produced by the null-function (WT-p.S269del concatemer) and negative GFP controls, indicating nonconducting KCNQ4 mutant channels (Fig. 2e). The linopirdine-sensitive potassium current of the WT-p.R331Q concatemer was slightly larger than that of the homomeric p.R331Q current, but with no significance, and the amplitude was far below the value of the WT-WT concatemer (Fig. 2e, f). Admittedly, some cells expressing the WT-p.R331Q concatemer showed a larger current than the WT-p.S269del concatemer and cells expressing GFP alone, which might be explained by individual cell-to-cell variability in membrane phospholipid levels. Taken together, these findings indicate that the novel pore-region variant p.A271_D272del exerts dominant-negative effects on the WT channel but that p.R331Q is a mutant protein for which some of its function can be restored when assembled with the functional WT protein under limited conditions. Cell surface expression analyses showed that these two KCNQ4 mutant proteins successfully localize to the plasma membrane and that their expression levels are comparable to that of the WT protein, excluding defective plasma membrane trafficking as the pathogenesis of these variants (Fig. S3).

### WT-p.G319D concatemers exhibit hyperactive channel activity with negative shifts in the voltage dependence of activation

**Fig. 3:**
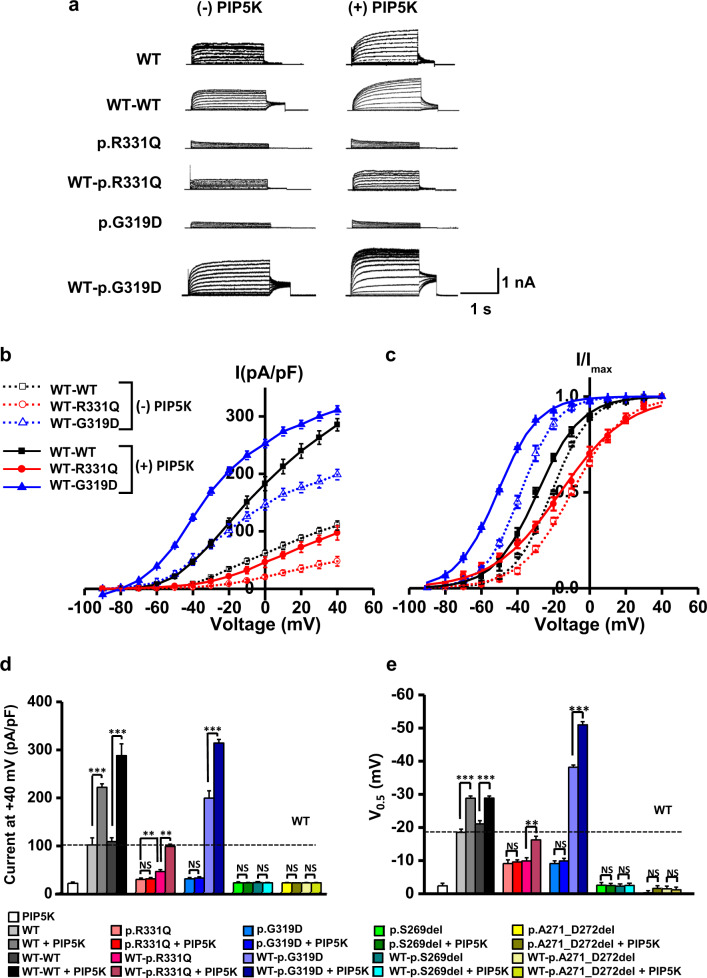
Rescue effects of the PIP5 kinase on KCNQ4 mutant channels. **a** The effects of phosphatidylinositol 4-phosphate 5-kinase (PIP5K) expression were compared between homomeric mutant channels (p.R331Q and p.G319D) and heteromeric tandem concatemer channels assembled from WT-p.R331Q and WT-p.G319D. Total K^+^ currents were measured in the absence (-PIP5K) or the presence of PIP5K expression (+PIP5K) in HEK293T cells. **b** Comparison of I-V curves between homomeric and tandem concatemer channels assembled from WT-WT, WT-p.R331Q, and WT-p.G319D. **c** Steady-state activation curves of the homomeric and tandem concatemer channels are compared (*n* = 10–12). **d, e** Comparison of K^+^ current densities measured at +40 mV and half-activation voltages (V_0.5_). WT-p.R331Q and WT-p.G319D concatemer mutant channels, but not homomeric mutant channels, were activated by PIP5K **d**, with a concomitant shifting of activation curve to negative voltage ranges **e**. Two pore-region mutant channels (p.S269del and p.A271_D272del) were unresponsive to PIP5K expression. The horizontal dotted lines in the graphs indicate the values obtained from homomeric WT channels (WT). The names of the groups are indicated in insets on the graph. Mean ± SEM (*n* = 10–12); ^**^P < 0.01, ^***^P < 0.005; NS, not significant.

The potassium currents of the heteromeric p.G319D channels were larger than those of homomeric WT channels when the cells expressed both the WT and p.G319D mutant proteins (3:1 and 2:2 ratios; Fig. S2b). Notably and unexpectedly, a significant difference in the mean value of linopirdine-sensitive currents was found for the WT-p.G319D concatemer (174.9 ± 51.5 pA/pF at +40 mV, *P* < 0.05) compared with that of WT-WT concatemers (95.2 ± 10.4 pA/pF; Fig. 2f). In another set of experiments, the total potassium current of the WT-p.G319D concatemer (198.7 ± 15.0 pA/pF at +40 mV) was approximately twice the amplitude of the WT-WT concatemer (110.2 ± 7.8 pA/pF) (Fig. 3a). Additionally, the WT-p.G319D concatemer showed a marked shift in the current-voltage (I-V) relationship (Fig. 2f and Fig. 3b) and the voltage dependence of activation toward negative potentials. The calculated half-activation voltage (V_0.5_) of the WT-p.G319D concatemer was −38.2 ± 0.6 mV (V_0.5_ of the WT-WT concatemer = −21.1 ± 1.0 mV) (Fig. 3c, e). Based on these results, we concluded that p.G319D did not exert a dominant-negative inhibitory effect on WT channels, unlike the other novel variants, but that it is the first hypermorphic DFNA2 variant with a gain of function when heterotetramerized with WT KCNQ4 subunits. In addition, cell surface expression analyses showed that both mutant KCNQ4 (p.G319D and WT-p.G319D concatemer) and WT proteins localized to the plasma membrane (Fig. S4).

### Rescue effects of the PIP5 kinase and KCNQ activators on the impaired potassium currents of novel *KCNQ4* variants

**Fig. 4:**
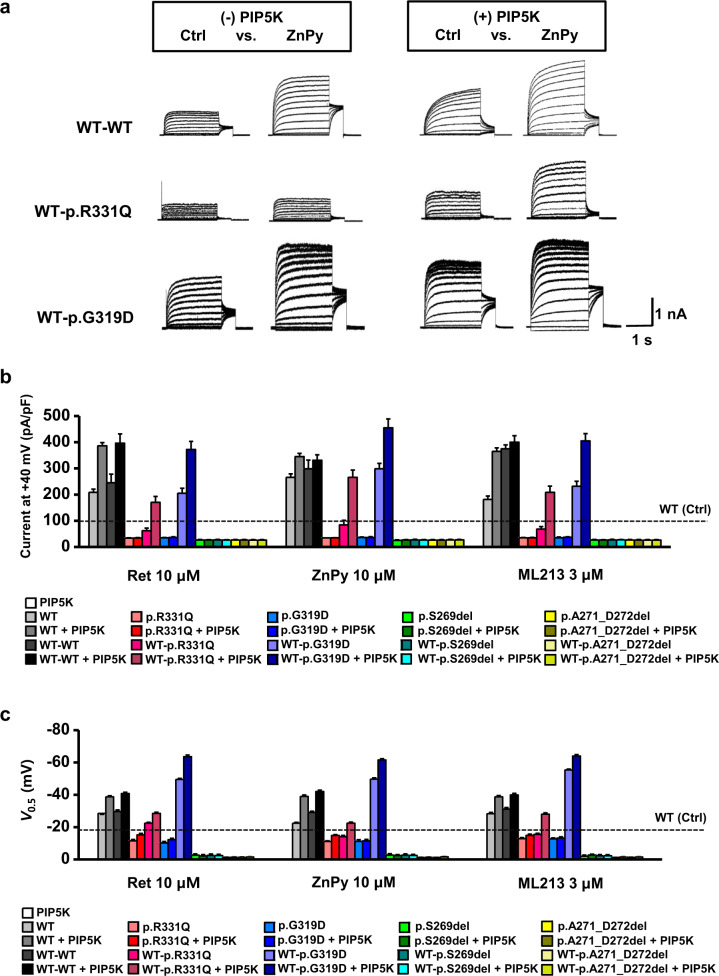
Rescue effects of KCNQ openers on KCNQ4 mutant channels assembled from tandem concatemers. **a** Total K^+^ currents were measured in the absence (-PIP5K) or presence (+PIP5K) of PIP5K expression in HEK293T cells, and responses to KCNQ openers were recorded. Representative current traces recorded before (Ctrl) and after 10 μM ZnPy treatment are presented for each concatemer channel. **b** Rescue effects of KCNQ openers (10 μM Ret, 10 μM ZnPy, and 3 μM ML213) on KCNQ4 mutant channels assembled from homomeric settings or heteromeric tandem concatemers were compared in the absence or presence of PIP5K expression. Current densities measured at +40 mV were compared as bar graphs. **c** Half-activation voltages (V_0.5_) were calculated from activation curves, and the values are plotted as bars. Horizontal dotted lines in the graphs indicate the current density level **b** or V_0.5_ values **c** of the control homomeric WT channels (WT, Ctrl) measured in the absence of PIP5K and KCNQ openers. The names of the groups are indicated in insets on the graph. Mean ± SEM. *n* = 10–12.

Rescue effects of the PIP5 kinase and KCNQ activators on current density (pA/pF at +40 mV) and half-activation voltages (V_0.5_, act, mV) measured from the KCNQ4 channels were shown in Table S1 and Table S2, respectively. Increasing the PIP_2_ concentration by expressing PIP5K enhanced WT and WT-WT channel currents by more than twofold (Fig. 3b, d), with a negative shift in V_0.5_ values by approximately −10 mV (Fig. 3c, e). With PIP5K expression, KCNQ channel openers (Ret 10 µM, ZnPy 10 µM, and ML213 3 µM) further enhanced channel currents in an additive fashion, but no clear potentiating effect was observed after combination treatments (Fig. 4). These data suggest that WT and WT-WT KCNQ4 channels retain PIP_2_ sensitivity and are responsive to KCNQ openers. In sharp contrast, two pore-region variants (p.S269del and p.A271_D272del) were completely nonrescuable after treatments with PIP5K, KCNQ channel openers, or a combination of both, even in the form of heteromeric concatemers (WT-p.S269del and WT-p.A271_D272del) (Figs. 3 and 4).

The impaired conductance of homomeric p.R331Q mutant channels was not rescued by manipulations using PIP5K, KCNQ channel openers, or a combination of both (Figs. 3 and 4), whereas PIP5K, KCNQ openers, or a combination of both had an apparent rescue effect on heteromeric WT-p.R331Q concatemers with corresponding negative V_0.5_ shits (Figs. 3,4). Moreover, PIP5K expression alone recovered the conductance of WT-p.R331Q concatemer with negative V_0.5_ shifts, making it comparable to the current of the normal homomeric WT or WT-WT concatemer channel (Fig. 3b–e). Even without PIP5K expression, three KNCQ openers (10 μM Ret, 10 μM ZnPy, and 3 μM ML213) significantly increased the conductance of WT-p.R331Q concatemers by up to ~70% of the normal WT current (Fig. 4a, b). With PIP5K expression, activation of the WT-p.R331Q concatemer with KCNQ openers was prominent, and the current reached the level of normal WT or WT-WT currents measured in the absence of PIP5K (Fig. 4b, c). Therefore, we concluded that pathogenic heteromeric WT-p.R331Q channels can be rescued by an increased PIP_2_ concentration, treatment with KCNQ openers, or both.

Although nonconducting homomeric p.G319D mutant channels were not rescued by PIP5K or KCNQ openers or their combination (Figs. 3,4), hyperactive WT-pG319D concatemer channels were responsive to PIP5K or KCNQ openers, or both (Figs. 3, 4). Similar to WT-WT and WT-p.R331Q concatemers, the increase in WT-p.G319D concatemer currents by PIP5K or KCNQ openers was proportional to the degree of negative V_0.5_ shifts, suggesting that PIP_2_ activation is mediated by the facilitation of voltage-dependent channel gating.

**Fig. 5:**
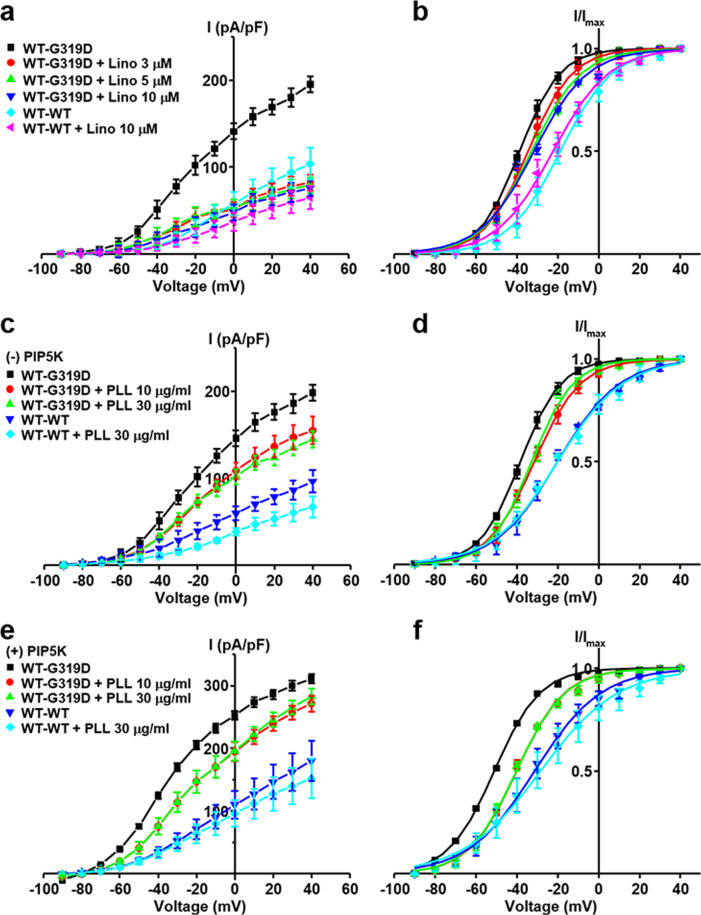
Downregulation of enhanced channel activity of WT-p.G319D tandem concatemers by a KCNQ inhibitor or screening of PIP_2_. Linopirdine (3–10 μM) inhibition of WT-p.G319D concatemer current is presented with I-V curves **a** and activation curves **b**. In the absence **c, d** or presence **e, f** of PIP5K expression, intracellularly applied poly-L-lysine (PLL, 10 or 30 μg/ml) attenuated the WT-p.G319D concatemer current **c, e**, with a concomitant shift of activation curves toward positive potentials **d, f**. The reducing effects of linopirdine and PLL on the WT-p.G319D channel current (*n* = 10) were compared with the effects on the WT-WT current (*n* = 6). Mean ± SEM.

The therapeutic strategy for a hyperactive WT-pG319D channel is a reduction in channel activity to a normal WT level. To this end, we examined the reducing effects of a KCNQ inhibitor and PIP_2_-chelating agent on the WT-pG319D channel current. The known KCNQ inhibitor linopirdine was able to reduce the hyperactive WT-p.G319D-mediated current to the WT-WT level, with a slight V_0.5_ shift toward a positive potential (Fig. 5a, b). With or without PIP5K expression conditions, interference of PIP_2_ activation in the WT-G319D channel by intracellularly applied polycation poly-L-lysine (PLL, 10 or 30 μg/ml) caused a significant reduction in the current, with a concomitant V_0.5_ shift to the right (Fig. 5c–f). Nonetheless, removing PIP_2_ alone did not completely restore the increased channel activity to WT-WT levels. The PLL-treated cells showed a 35% higher potassium current than WT concatemer channels, and V_0.5_ shifted 10 mV toward a positive potential. Therefore, the KCNQ inhibitor was more effective in reducing the channel current, and PIP_2_ chelation was more effective in normalizing the voltage dependence of channel activation. Overall, these results suggest that the enhanced channel conductance of p.G319D under heteromeric conditions can be downregulated *via* a KCNQ inhibitor (linopirdine), suppression of PIP_2_ activation, or combination therapy.

## Discussion

In this study, we explored the application of genotype/pathophysiology-based customized pharmacology to functionally rescue impaired KCNQ4-mediated potassium currents related to novel DFNA2 variants. Our two novel missense mutant channels (p.R331Q and p.G319D) responded to PIP5K- and KCNQ-regulating drug treatment, but only when the variants were assembled with WT KCNQ4 subunits. Specifically, application of PIP5K, channel openers, or a combination of both, if not to the same degree, restored the ion conductance of the p.R331Q variant in the forced heteromeric state compared with the lack of response to any of these treatments in the homomeric state. Furthermore, the development of a customized pharmacological approach for DFNA2 variants in this study is especially fueled by the identification of the first hypermorphic DFNA2 variant, p.G319D, and this variant may be amenable to KCNQ channel inhibitors and PIP_2_ suppression to regulate excessive potassium outflow. Thus, our results suggest the potential for tailored therapeutics for KCNQ4 variants and contribute to future clinical practices with respect to DFNA2.

Previous studies have demonstrated that even a single-nucleotide change close to the pore region may drastically impede ion permeation^1^, rendering channels nonfunctional, independent of channel gating^12^. Similarly, the two KCNQ4 pore-region variants identified in our study produced no detectable potassium currents and were completely nonrescuable by the application of PIP5K, channel openers, or both. In contrast, channel openers were previously found to partially restore the activity of heterogeneous KCNQ4 channels containing pore variant subunits; however, this mitigation of dominant-negative inhibitory effects may have relied exclusively on the potentiation of residual homomeric WT channel function^9,12^. Indeed, expression of homogenous forced heteromeric channels (i.e., tandem concatemers) was insensitive to pharmacological treatments, which resembles the previously reported unresponsiveness of WT:W276S or WT:G285S concatemers to a combination of channel openers^12^. Collectively, the lack of effect of pharmacological treatment on channels containing the pore-region variants identified in ours and previous studies may be attributed to either the lack of residual homomeric WT channels in the concatemer status or the inability of residual homomeric WT channels to respond to channel openers, even under PIP5K-enhanced conditions, or both. Thus, further exploration of novel therapeutics to potentiate currents through totally abolished KCNQ4 channels due to pore-region DFNA2 variants is warranted, though such efforts with a focus on the enhancement of the specificity of KCNQ4 openers have already been initiated^28^.

Channels containing a novel p.R331Q variant showed impaired potassium currents not only in homotetrameric states but also in heterotetrameric states, indicating its inhibitory mechanism on the pathogenesis of DFNA2. Of note, variants affecting basic and charged amino acids of the proximal C-terminus in most KCNQ channels are associated with various human diseases^13^. Moreover, variants in the proximal C-terminus of KCNQ channels have been shown to significantly attenuate affinity for PIP_2_^29^, leading to the decoupling of the voltage-sensing domain (VSD) with the pore domain (PD) and resulting in the failure of pore opening. Indeed, the residues around Arg331 in the KCNQ4 channel are well conserved and are predicted to be a target region for PIP_2_ binding^30,31^. Expectedly, the interaction between PIP_2_ and the Arg331 residue of KCNQ4 was supported by Protein Data Bank (Fig. S5). Furthermore, KCNQ1 double variants (Lys358Ala/Arg360Ala), located at the equivalent position of the proximal C-terminal residue (Arg331) of KCNQ4, show no ion conductance due to disrupted PIP_2_ binding affinity, leading to long QT syndrome^32^. Based on these results, the null activity of KCNQ4 channels containing p.R331Q is likely due to an altered electrostatic interaction with PIP_2_ and subsequent abolishment of the ion conductance *via* impaired coupling with the PD and/or VSD.

Notably, the present study demonstrated that forced heteromerization of p.R331Q with the WT channel, which mimics the heterozygous condition in vivo, induced recovery of impaired channel activity through PIP5K expression but that tandem concatemers of the WT and pore-region variant were nonrescuable even at the same high PIP5K expression. This suggests that disrupted PIP_2_ binding sites due to p.R331Q could be somehow related to the pathophysiology of the loss of function of mutated KCNQ4 channels containing p.R331Q. Our hypothesis is partly corroborated by previous work by Soldovieri *et al*.^33^, who reported that PIP5K enables a homomeric KCNQ2 mutant channel containing a variant of Arg325 residue that is equivalent position to the Arg331 of KCNQ4 to potentiate potassium currents. Regardless, it remains elusive why the current is not restored by cotransfection of PIP5K with homomeric p.R331Q KCNQ4 channel plasmids. Nonetheless, PIP_2_- or KCNQ opener-induced activation of WT-p.R331Q channels might be explained by the action of these molecules on functional WT subunits, irrespective of the underlying pathogenic mechanism of p.R331Q^9,12^. Unlike for p.R331Q, the more drastic dominant-negative effect of pore variants on the WT subunit precluded the efficacy of PIP_2_ and KCNQ openers because the variants result in the collapse of ion permeation sites.

**Fig. 6:**
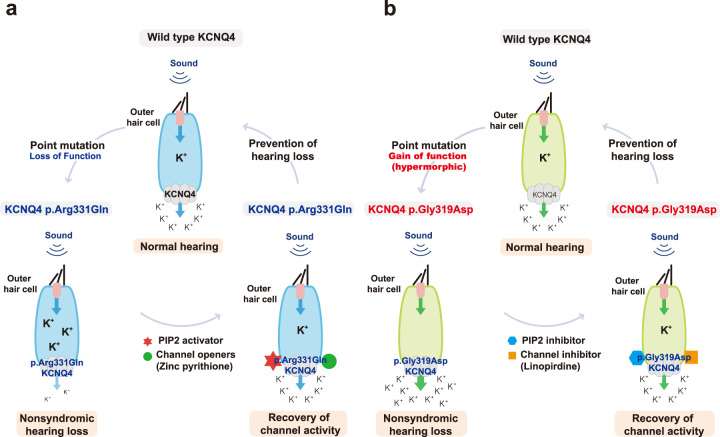
Schematic illustration of different pathophysiological mechanisms of KCNQ4 variants on DFNA2. **a** Loss-of-function caused by KCNQ4 p.R331Q and its restoration following phospholipid phosphatidylinositol 4,5-bisphosphate (PIP_2_) activator (i.e., PIP5K) and channel openers. **b** Gain of function caused by KCNQ4 p.G319D and its restoration following interference of PIP_2_ activation by intracellular application of a polycation poly-L-lysine (PIP_2_ inhibitor) and channel inhibitor.

It should also be noted that ZnPy has been reported to rescue KCNQ channel activity in the absence of PIP_2_^34^, even though retigabine acts as a primary modifier of gating kinetics by stabilizing the conformation of pore and voltage sensors. In addition, potentiation of WT-p.R331Q concatemer channel activity by a combination of ZnPy and PIP5K reached a similar level to that of the ZnPy and PIP5K-applied homomeric WT channel (Fig. 4b), which was not observed with the combination of retigabine with PIP5K. In accordance, stronger potentiation of KCNQ4-mediated potassium currents in OHCs was previously demonstrated when retigabine was combined with ZnPy^12^. Therefore, retigabine and ZnPy may not only complement each other but also exert synergistic effects when combined with outstripped WT channel activity in terms of potentiating defective KCNQ4-mediated potassium currents. Our results are clinically encouraging because channel openers significantly restored the channel activity of forced homogenous heteromeric channels (WT-p.R331Q) that mimic the heterozygous condition in vivo, particularly when a high concentration of PIP_2_ was maintained (Fig. 6a).

Another novel missense variant, p.G319D, which is located in the C-terminal part of the transmembrane S6 segment, produced a nonconducting KCNQ4 channel in a homologous setting, suggesting that it acts as a pathogenic variant. The Gly348 residue in KCNQ1, corresponding to Gly319 in KCNQ4, is critical to channel gating, as alteration of the residue produced a nonconducting KCNQ1 channel^35^. A GSG motif containing a Gly348 residue and a highly conserved glycine residue is located in the C-terminal part of S6^35,36^. The GSG motif of KCNQ1 specifically interacts with the S4−S5 linker and the S5 segment^37^, playing a pivotal role in the opening and closing of the channel gate. Experimental substitution of Gly348 with an alanine residue (G348A) shifts the voltage dependence of KCNQ1 channel activation by 25 mV toward a negative potential in homomeric settings, whereas G348W fails to generate any potassium current^35^. These observations are somewhat consistent with our observations, whereby WT-p.G319D shifted voltage-dependent activation by approximately 20 mV to a negative potential compared with homomeric WT channels (V_0.5_; −38.2 ± 0.5 mV vs. −18.5 ± 1.0 mV). Our findings, together with the study in KCNQ1^35^, suggest that the C-terminal part of S6 with the GSG motif plays a pivotal role in regulating KCNQ4 channel gating and kinetics. Interestingly, the homomeric mutant channel containing p.G319D was completely unresponsive to channel openers, though the current from a homomeric channel containing p.G321S was substantially restored by the combined application of both channel openers. These two variants are located in similar topologies while providing enigmatic disparity. The difference in sensitivity between the two variants to channel openers may depend on the amino acid substituted and the size of the channel current involved in gating.

Importantly, we observed that the forced homogenous heterotetrameric WT-p.G319D concatemer produced comparable—or even increased—potassium currents compared with the WT-WT concatemer. To the best of our knowledge, this is the first hypermorphic *KCNQ4* variant reported. Our data indicate that the dominant-negative inhibitory effect does not underlie the pathogenesis of DFNA2 related to p.G319D. Considering that activation of residual homomeric WT channels is key in determining the pharmacological responsiveness of the tetrameric *KCNQ4* variant channel^12^, the WT-p.G319D concatemer, which is presumably free from a dominant-negative effect, is expected to retain more channel activity than other heteromeric mutant channels, showing dominant-negative inhibition. A previous study demonstrated different gating mechanisms in the KCNQ1 channel between the two variants p.G348W and p.G348A. The p.G348A variant affects the gating properties of the KCNQ1 channel in such a way that it shifts the voltage dependence of activation toward a negative potential, which is associated with the open state^35^. Similarly, the p.G319D variant may contribute to the stabilization of gating kinetics in a sustained open state if assembled as a heterotetrameric channel combined with the WT protein, as in the heterozygous condition. It is easily conceivable that defective plasma membrane trafficking would not be the main pathogenic mechanism of the loss-of-function caused by p.G319D, as based on the robustly enhanced channel activity observed with heterogeneous KCNQ4 channels and immunofluorescence experiments of the mutant KCNQ4 protein (p.G319D) (see Fig. S4). Instead, a sustained open state of the KCNQ4 channel predicted from p.G319D-mediated hyperactivation of gating may lead to oversecretion of K^+^ through the KCNQ4 channel from OHCs, potentially leading to K^+^ depletion in these cells. Furthermore, loss of intracellular K^+^ in OHCs may elicit chronic hyperpolarization, possibly affecting Ca^2+^ influx through voltage-gated Ca^2+^ channels or Ca^2+^-modulated proteins (calmodulin) and causing their subsequent degeneration due to chronic cellular stress. Given that K^+^ is the major charge carrier for sensory transduction in the inner ear^38^, chronic K^+^ depletion may hinder its proper recycling and trigger neurotransmission for the process of hearing. This, in turn, suggests that the hypermophic p.G319D variant may cause hearing loss. Although K^+^ depletion in OHCs due to KCNQ4 overactivation may be a somewhat new finding in the literature, hypokalemia per se might be a manifestation of various diseases^39^, making hypermorphic *KCNQ4* variants clinically important.

From this perspective, therapeutic agents that ameliorate KCNQ4 channel activity while maintaining sufficient current levels may allow for rescue of the DFNA2 phenotype due to the hypermorphic KCNQ4 variant (Fig. 6b). We, for the first time, demonstrated that a KCNQ inhibitor and PIP_2_ interference could potentially stabilize the enhanced potassium currents of the WT-p.G319D concatemer in transfected cells to a similar level as the WT-WT concatemer, suggesting a potential therapeutic strategy for yet-to-be-identified hypermorphic KCNQ variants. We believe that the development of knock-in mice expressing this hypermorphic KCNQ4 variant would provide an in vivo tool to address in-depth questions and establish therapies for hypermorphic KCNQ variants^39^.

In this study, we observed diverse audiogram configurations not limited to orthodox high-frequency specific hearing loss. In particular, nonpore-region KCNQ4 variants were associated with nontypical high-frequency hearing loss, though the small sample number precludes any correlation between domain and phenotype. A possible correlation between auditory phenotypes, such as the onset of disease and affected frequencies, and genotypes (i.e., missense vs. deletion) has been proposed^6,40^. Nevertheless, additional studies are necessary to draw any meaningful conclusions. It appears that differential spatiotemporal expression of KCNQ4 in spiral ganglion cells and inner hair cells may account for highly complex auditory phenotypes^41–43^. Alternatively, the pathogenic effect of nonpore-region variants may vary, depending on which KCNQ4 isoforms are mainly affected by the variant, as each isoform may show a different tonotopic distribution. Complex alternative splicing of human KCNQ4 isoforms may further diversify the functional consequences of *KCNQ4* variants in terms of auditory phenotypes^44^.

## Supplementary information

Novel KCNQ4 variants residing in different functional domains confer genotype and mechanism-based therapeutics in patients with nonsyndromic hearing loss

## Data Availability

The data that support the findings of this study are available from the corresponding author upon reasonable request. Some data may not be made available because of privacy or ethical restrictions.
