# Supplementary material for: Novel KCNQ4 variants in different functional domains confer genotype- and mechanism-based therapeutics in patients with nonsyndromic hearing loss
Source: Exp Mol Med. 2021 Jul 28;53(7):1192–204. doi: 10.1038/s12276-021-00653-4 (PMC8333092; doi:10.1038/s12276-021-00653-4)
Supplement: Supplementary file 1 — Novel KCNQ4 variants residing in different functional domains confer genotype and mechanism-based therapeutics in patients with nonsyndromic hearing loss [file 12276_2021_653_MOESM1_ESM.pdf]

supplementary information

Fig S1. Schematic illustration of the KCNQ4 functional domain map.

Three novel KCNQ4 variants resided in different functional domains in the current study: p.A271_D272del located in the pore loop domain, p.G319D located in the C-terminal part of the transmembrane S6 segment, and p.R331Q located in the proximal cytoplasmic C-terminus. The capital letters in the squares refer to the conserved amino acids of the corresponding residue of the missense variants of KCNQ4 (e.g., F, phenylalanine). N, intracellular N-termini; C, intracellular C-termini; S1-S6; six transmembrane domains.

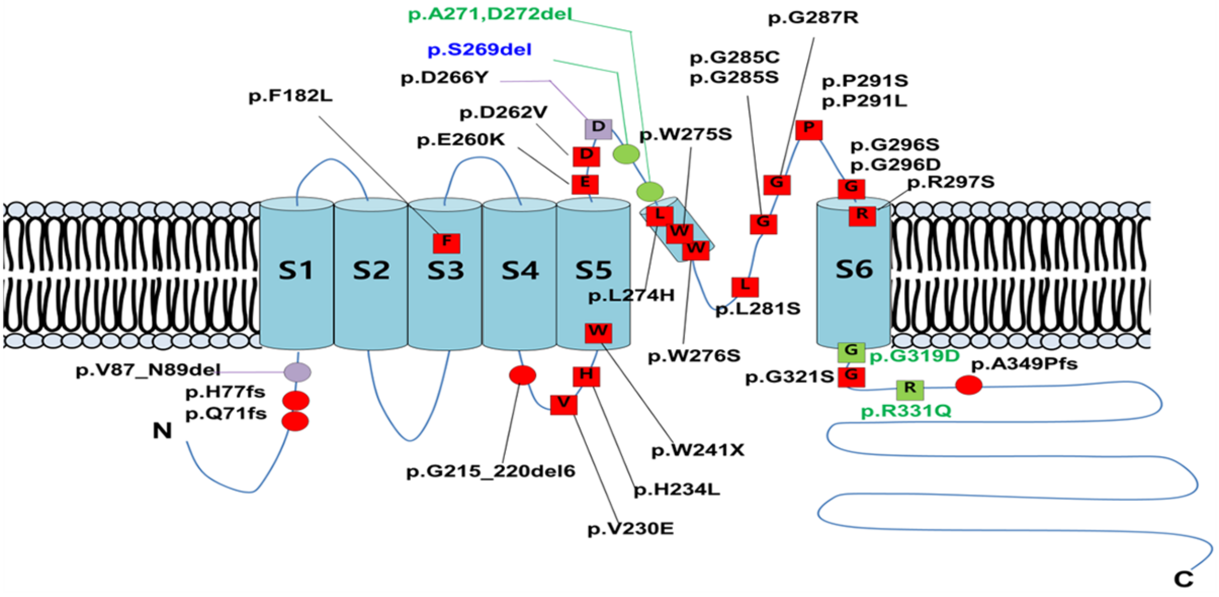

Fig S2. Dominant-negative effects of the KCNQ4 variant channels.

(**a**) Each of the KCNQ4 variants (p.S269del, p.A271_D272del, p.G319D, or p.R331Q) were co-expressed with WT KCNQ4 at the indicated WT:variant cDNA molar ratios, and linopirdine-sensitive K^+ currents were recorded at +40 mV. Dashed lines under the K^+ current traces indicate zero current levels at a holding potential of -80 mV. (**b**) Comparison of current densities (pA/pF) at +40 mV (n = 6–17). WT:variant cDNA ratios are indicated under the bar graphs, and the total amount of cDNA was equalized in all groups by adding empty pRK5 vector. (**c**) Suppression of WT-mediated current by the co-expression of KCNQ4 variant (var.) channels. The mean values of the current densities at +40 mV were normalized, and the current suppression ratios were plotted against WT / (WT + var.) cDNA molar ratios. Dashed line with a square symbol indicates the predicted suppression ratios expected for the tetramerized channel with a dominant negative subunit. Mean ± SEM

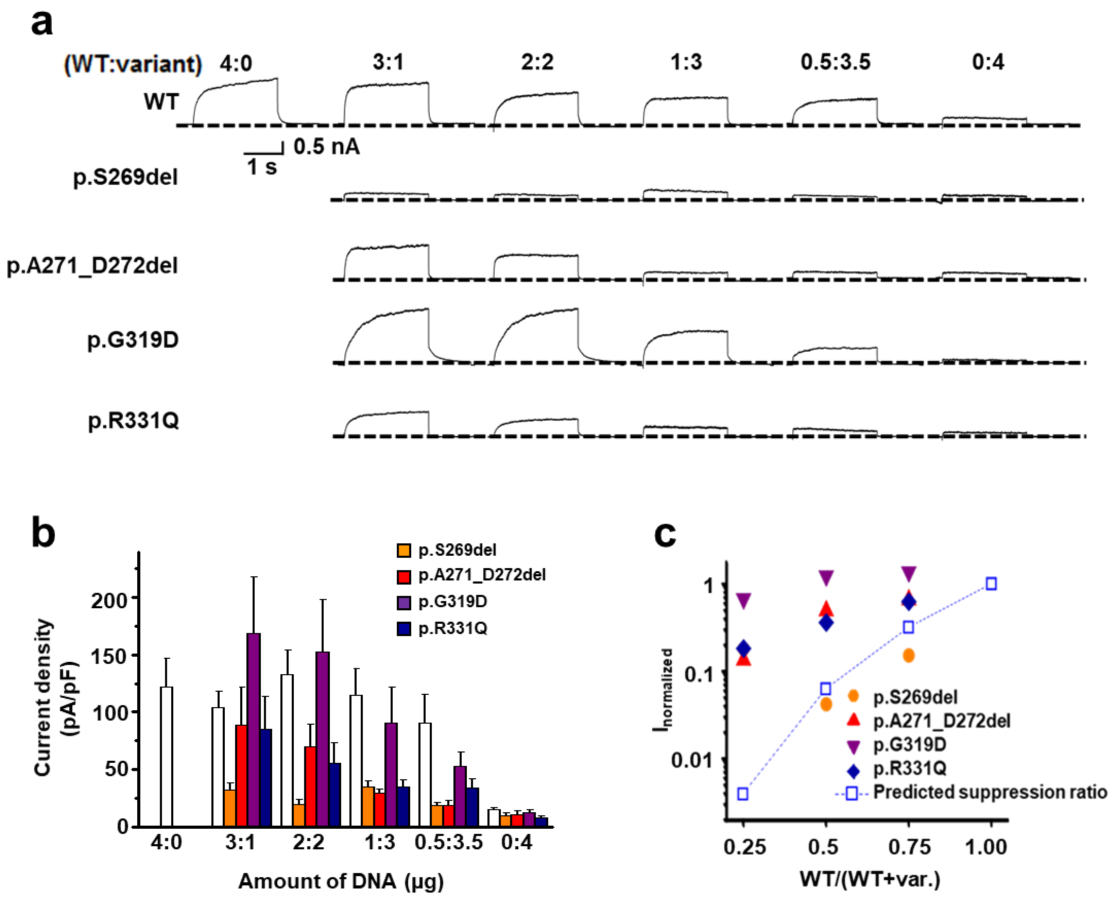

Fig S3. Functional testing of KCNQ4 p.A271_D272del, p.R331Q variants.

(a) Immunofluorescence of HEK293T cells transfected with N-terminally FLAG-tagged KCNQ4 WT, KCNQ4 p.A271_D272del, and KCNQ4 p.R331Q. Cells were immunostained with anti-FLAG (green) and anti-Concanavalin A (red, Invitrogen, CAT no.C860) antibodies. All mutant KCNQ4 proteins (p.A271_D272del and p.R331Q) and WT protein were observed on the plasma membrane (b) Surface biotinylation of HEK293T cells transfected with KCNQ4 WT, KCNQ4 p.A271_D272del, and KCNQ4 p.R331Q. ***, statistical significance (p<0.001).

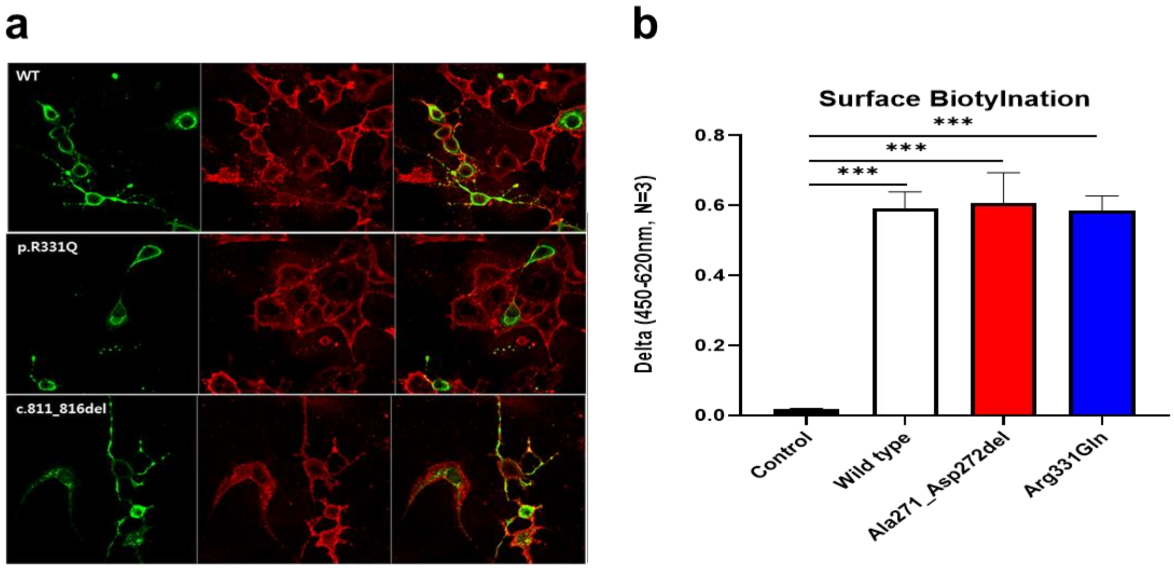

Fig S4. Immunofluorescence of wild-type and mutant p.G319D KCNQ4 proteins in COS-7 cells.

COS-7 cells were transfected with N-terminally Myc-tagged (green) wild-type (WT) and mutant KCNQ4 (p.G319D) clones. Cells were immunostained with anti-Myc and anti-Concanavalin A (red, Invitrogen, CAT no.C860) antibodies. Nuclei were stained with DAPI (blue). Concanavalin A binds to glycosylated proteins present in the plasma membranes, serving as a plasma membrane marker (red). As shown in Fig.S5, both mutant KCNQ4 proteins (p.G319D and WT-p.G319D concatemer) and WT protein were observed on the plasma membrane, giving rise to yellow signals.

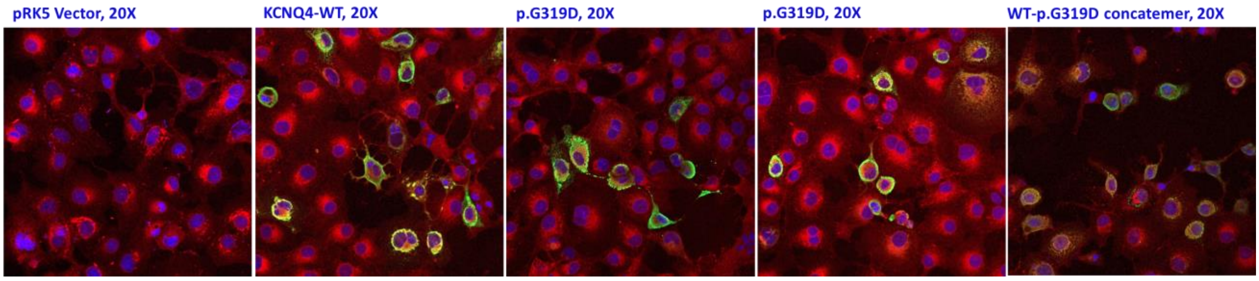

Fig S5. 3-Dimensional modeling based on crystal structure of 2r9r shows a likely interaction between PIP2 and arginine. 2r9r is the voltage-gated potassium channel of *Rattus norvegicus* encoded by KCNb3. Its amino acid sequences are homologous with that of KCNQ4. The crystal structure of 2r9r obtained from the Protein Data Bank is found to interact between PIP2 and arginine. This arginine corresponds with the Arg331 residue of KCNQ4 on alignment of the 2r9r structure.

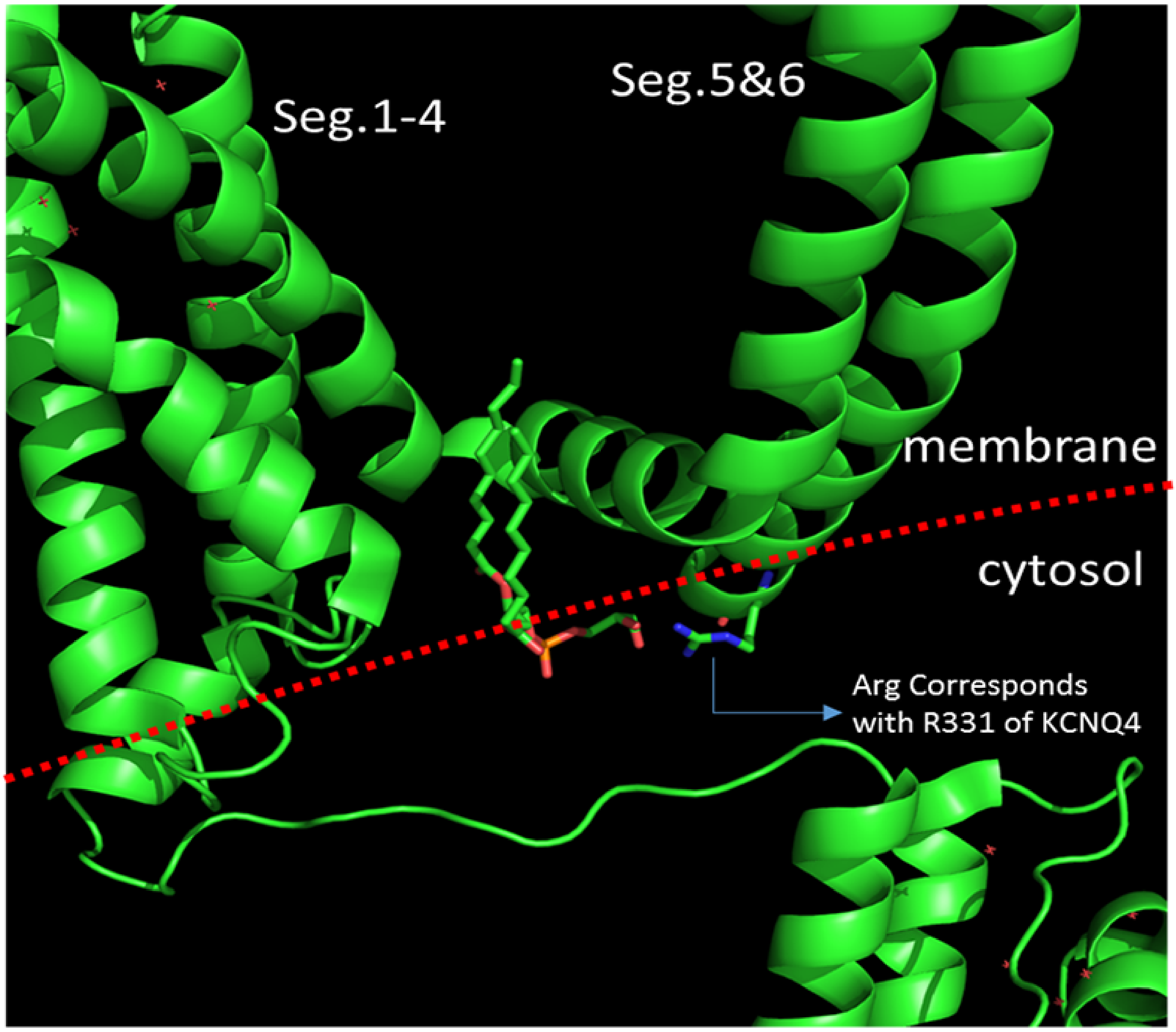

Table S1. Current density (pA/pF at +40 mV) measured from the KCNQ4 channels.

	Control	Ret (10 µM)	ZnPy (10 µM)	ML213 (3 µM)
**PIP_5K**	25 ± 2.8	26 ± 1.8	25 ± 1.3	25 ± 1.9
**WT**	103.3 ± 14.5	208.6 ± 13.0	265.7 ± 13.0	181.3 ± 13.0
**WT + PIP_5K**	220.3 ± 7.3***	386.5 ± 12.0	345.2 ± 12.0	365 ± 13.2
**WT-WT**	110.2 ± 7.8	245 ± 33	299 ± 30	375 ± 15
**WT-WT + PIP_5K**	285.4 ± 23.9***	396.4 ± 35	330.9 ± 21.2	399.9 ± 25
**p.R331Q**	33 ± 2.8	34 ± 1.1	34.5 ± 0.2	35 ± 1.3
**p.R331Q + PIP_5K**	34.2 ± 2.8	34.2 ± 1.5	35 ± 0.5	35.1 ± 1.5
**WT-p.R331Q**	49 ± 4.0	62.4 ± 9.5	84.3 ± 17.7	68.2 ± 9.7
**WT-p.R331Q + PIP_5K**	100.3 ± 4.7**	170.5 ± 22.5	265.7 ± 28	208.6 ± 23.8
**p.G319D**	34.1 ± 2.8	35 ± 1.1	36 ± 2.1	35.1 ± 3
**p.G319D + PIP_5K**	35.8 ± 2.8	36 ± 2.8	36.1 ± 3	37 ± 2
**WT-p.G319D**	198.7 ± 15	205 ± 19.2	299 ± 20.0	232 ± 19.6
**WT-p.G319D + PIP_5K**	311.0 ± 7.5***	372.5 ± 30.7	455 ± 34.2	405 ± 27.8
**p.S269del**	26.1 ± 2.0	26.3 ± 2.0	25.1 ± 2.1	26.3 ± 1.9
**p.S269del + PIP_5K**	26.1 ± 0.7	26.3 ± 1.4	26.5 ± 1.4	26.3 ± 1.5
**WT-p.S269del**	26.5 ± 2.0	26.8 ± 2.1	27.0 ± 2.0	27.0 ± 1.9
**WT-p.S269del + PIP_5K**	26.1 ± 0.8	26.3 ± 1.3	26.5 ± 1.2	27.1 ± 1.4
**p.A271_D272del**	26.4 ± 2.0	26.3 ± 2.0	26.2 ± 2.1	26.3 ± 1.9
**p.A271_D272del + PIP_5K**	26.2 ± 1.2	26.3 ± 2.1	27.1 ± 2.2	26.0 ± 2.0
**WT-p.A271_D272del**	26.3 ± 2.0	26.1 ± 1.9	26.8 ± 2.0	26.2 ± 1.8
**WT-p.A271_D272del + PIP_5K**	26.1 ± 2.0	26.2 ± 1.5	27.0 ± 1.7	25.9 ± 1.9

Control, non-treated cells; Ret, retigabine; ZnPy, zinc pyrithione; WT, wild type; PIP_5K, phosphatidylinositol 4-phosphate 5-kinase

(-) PIP_5K vs. (+) PIP_5K: $^{*}P < 0.05$, $^{**}P < 0.01$, $^{***}P < 0.005$.

Sample numbers (n) = 10-12 for each group.

Table S2. Half-activation voltages ($V_{0.5,act}$, mV) measured from the KCNQ4 channels.

	Control	Ret (10 µM)	ZnPy (10 µM)	ML213 (3 µM)
**PIP5K**	-2.4 ± 0.8	-2.4 ± 0.6	-2.3 ± 0.7	-2.4 ± 0.8
**WT**	-18.5 ± 1.0	-28.5 ± 1.0$^{\#\#}$	-22.4 ± 0.6$^{\#\#}$	-28.3 ± 0.8$^{\#\#}$
**WT + PIP5K**	-28.8 ± 0.7***	-38.7 ± 0.8$^{\#\#}$	-39.0 ± 0.9$^{\#\#}$	-38.7 ± 0.9$^{\#\#}$
**WT-WT**	-21.1 ± 1.0	-29.7 ± 0.9$^{\#\#}$	-29.1 ± 0.8$^{\#}$	-31.1 ± 1.0$^{\#\#}$
**WT-WT + PIP5K**	-28.9 ± 0.7***	-40.8 ± 0.8$^{\#\#}$	-42.0 ± 0.9$^{\#\#}$	-39.9 ± 1.0$^{\#\#}$
**p.R331Q**	-9.0 ± 1.1	-11.5 ± 0.8	-11.1 ± 0.5	-12.8 ± 0.7
**p.R331Q + PIP5K**	-9.6 ± 0.7	-15.2 ± 0.9$^{\#}$	-14.8 ± 0.6	-14.9 ± 0.8
**WT-p.R331Q**	-9.9 ± 1.0	-22.4 ± 0.6$^{\#\#}$	-14.1 ± 0.8$^{\#\#}$	-15.5 ± 0.7$^{\#\#}$
**WT-p.R331Q + PIP5K**	-16.3 ± 1.1**	-28.5 ± 0.7$^{\#\#}$	-22.4 ± 0.7$^{\#\#}$	-28.0 ± 0.8$^{\#\#}$
**p.G319D**	-9.1 ± 0.8	-10.2 ± 0.9	-11.2 ± 1.0	-12.7 ± 0.7
**p.G319D + PIP5K**	-9.9 ± 0.8	-12.2 ± 0.9	-11.5 ± 0.9	-13.2 ± 0.8$^{\#}$
**WT-p.G319D**	-38.2 ± 0.6	-49.5 ± 0.7$^{\#\#}$	-49.7 ± 0.8$^{\#\#}$	-55.4 ± 0.6$^{\#\#\#}$
**WT-p.G319D + PIP5K**	-51.0 ± 1.0***	-63.6 ± 1.0$^{\#\#}$	-61.6 ± 0.8$^{\#\#}$	-64.0 ± 0.9$^{\#\#\#}$
**p.S269del**	-2.6 ± 0.8	-2.6 ± 0.9	-2.7 ± 0.9	-2.0 ± 0.7
**p.S269del + PIP5K**	-2.5 ± 0.7	-2.2 ± 0.8	-2.4 ± 0.6	-2.7 ± 0.6
**WT-p.S269del**	-2.3 ± 0.7	-2.5 ± 0.9	-2.6 ± 0.9	-2.3 ± 0.7
**WT-p.S269del + PIP5K**	-2.5 ± 0.6	-2.4 ± 0.8	-2.5 ± 0.6	-2.3 ± 0.6
**p.A271_D272del**	-1.9 ± 0.7	-2.0 ± 0.5	-1.8 ± 0.6	-1.7 ± 0.5
**p.A271_D272del + PIP5K**	-1.8 ± 0.8	-1.7 ± 0.6	-1.9 ± 0.5	-1.7 ± 0.6
**WT-p.A271_D272del**	-1.7 ± 0.8	-.1.6 ± 0.5	-1.7 ± 0.4	-1.9 ± 0.3
**WT-p.A271_D272del + PIP5K**	-1.8 ± 0.8	-1.7 ± 0.4	-1.6 ± 0.4	-1.7 ± 0.5

Control, non-treated cells; Ret, retigabine; ZnPy, zinc pyrithione; WT, wild type; PIP_5K, phosphatidylinositol 4-phosphate 5-kinase

(-) PIP_5K vs. (+) PIP_5K: $^{*}P < 0.05$, $^{**}P < 0.01$, $^{***}P < 0.005$.

Control *vs*. KCNQ activators: $^{\#}P < 0.05$, $^{\#\#}P < 0.01$, $^{\#\#\#}P < 0.005$

Sample numbers (n) = 10-12 for each group.
